# Overexpression of CD44v8-10 in Colon Polyps—A Possible Key to Early Diagnosis

**DOI:** 10.3389/pore.2021.614281

**Published:** 2021-03-30

**Authors:** Milan Dastych, Frantisek Hubatka, Pavlina Turanek-Knotigova, Josef Masek, Radek Kroupa, Milan Raška, Jaroslav Turanek, Lubomir Prochazka

**Affiliations:** ^1^ Department of Gastroenterology and Internal Medicine, University Hospital Brno and Faculty of Medicine Masaryk University Brno, Brno, Czech Republic; ^2^ Department of Pharmacology and Toxicology, Veterinary Research Institute, Brno, Czech Republic; ^3^ C2P NEXARS, Campus Science Park, Brno, Czech Republic; ^4^ Department of Immunology, Faculty of Medicine and Dentistry, Palacky University Olomouc, Olomouc, Czech Republic; ^5^ Faculty of Medicine in Hradec Kralove, Institute of Hygiene and Preventive Medicine, Charles University, Hradec Kralove, Czech Republic; ^6^ Institute of Physics of the Czech Academy of Sciences, Prague 8, Czech Republic

**Keywords:** CD44 isoforms, colorectal precancerosis, colon polyps, cancer markers, RNA splicing

## Abstract

**Background and aims:** The majority of colorectal cancers arise from detectable adenomatous or serrated lesions. Here we demonstrate how deregulated alternative splicing of CD44 gene in diseased colon mucosa results in downregulation of standard isoform of CD44 gene (CD44s) and upregulation of variant isoform CD44v8-10. Our aim is to show that upregulation of CD44v8-10 isoform is a possible marker of precancerous lesion in human colon.

**Methods:** We analysed pairs of fresh biopsy specimen of large intestine in a cohort of 50 patients. We studied and compared alternative splicing profile of CD44 gene in colon polyps and adjoined healthy colon mucosa. We performed end-point and qRT PCR, western blotting, IHC staining and flow cytometry analyses.

**Results:** We detected more than five-fold overexpression of CD44v8-10 isoform and almost twenty-fold downregulation of standard isoform CD44s in colon polyps compared to adjoined healthy tissue with *p* = 0.018 and *p* < 0.001 in a cohort of 50 patients. Our results also show that aberrant splicing of CD44 occurs in both biologically distinct subtypes of colorectal adenoma possibly in ESRP-1 specific manner.

**Conclusion:** 92% of the colon polyp positive patients overexpressed CD44v8-10 isoform in their colon polyps while only 36% of them had positive fecal occult blood test which is currently a standard non-invasive screening technique.

**Impact:** We believe that our results are important for further steps leading to application of CD44v8-10 isoform as a biomarker of colorectal precancerosis in non-invasive detection. Early detection of colon precancerosis means successful prevention of colorectal carcinoma.

## Introduction

Colorectal cancer (CRC) is the second leading cause of cancer related death in Europe [1] and fourth and third in males and females worldwide [2]. The basic mechanism of colorectal tumorigenesis is quite straightforward stepwise process from precancerous to malignant stage [3, 4] although molecular processes during tissue remodeling and tumor microenvironment interaction remain under intensive research. CRC is preventable partly due to early detection of preneoplastic or neoplastic lesions in the large intestine unlike most of other cancers. Regular screenings mainly colonoscopies and fecal occult blood testing significantly increased the ratio of early detected CRC patients and proved to be effective [5]. Possible early detection of precancerous lesions through CD44 receptor became the focus of our study. CD44 receptor is expressed widely throughout the human body in both standard and variable isoforms [6]. CD44 receptor has several physiological functions like cell adhesion through binding of hyaluronic acid, the main component of extracellular matrix [7, 8] and participation in signal transduction and apoptosis control [9].

**FIGURE 1:**
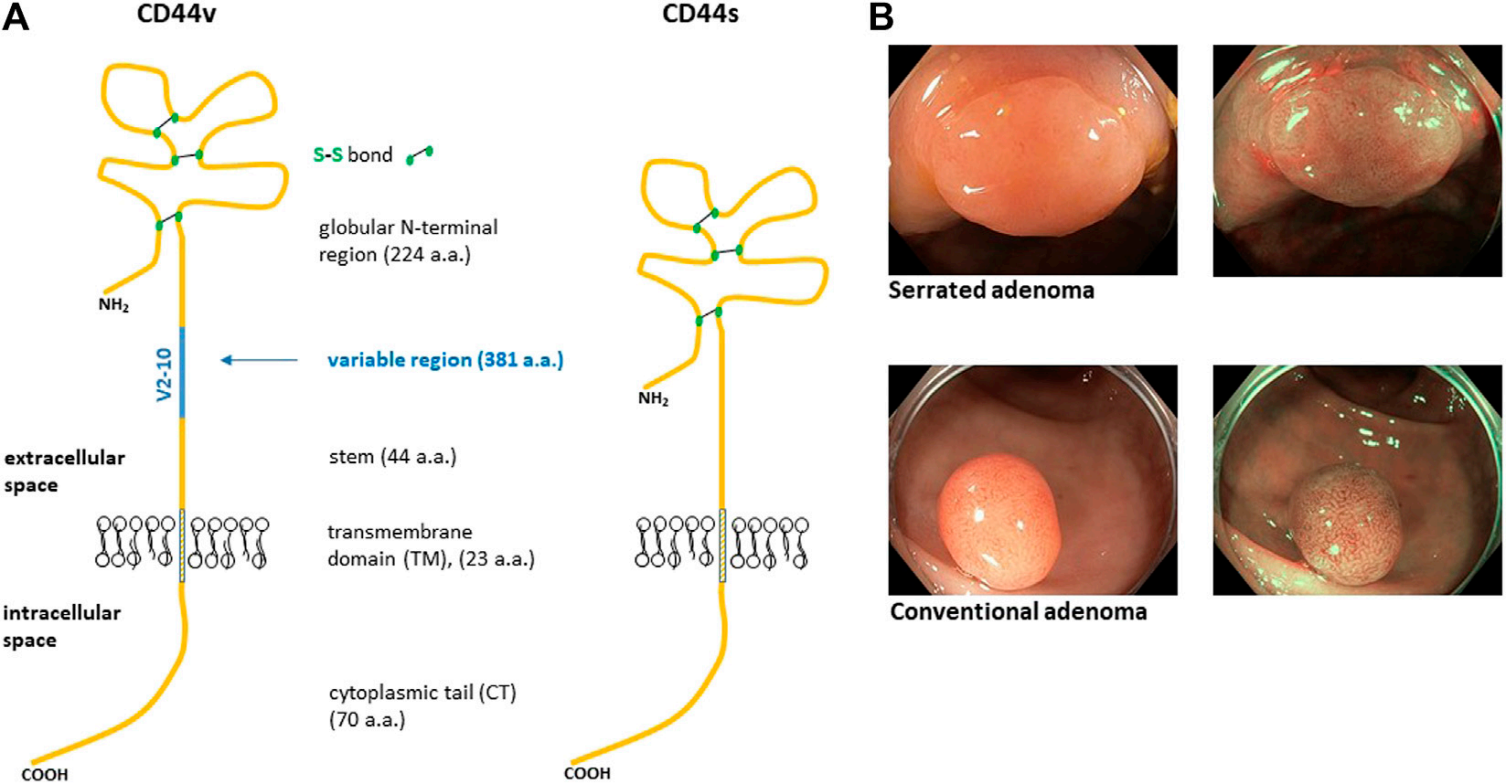
Structure of human CD44 protein **(A)**. The blue line represents alternative region that is completely missing in CD44s isoform. Variable region of CD44 is a site of heavy O-glycosylation. The globular amino terminal domain of CD44 contains three disulfide bonds and two hyaluronan binding motifs [6]. **(B)** Representative endoscopic images of colon adenomas we sampled. The top image displays serrated adenoma while the bottom image displays conventional adenoma. The images are taken in two modes, i.e., white light and narrow band image (NBI).

Since 1990’s it was discovered that CD44 transcript is a subject of alternative splicing that generates various isoforms of CD44 (Figure 1A). Alternative splicing of CD44 is tissue specific and often deregulated in cancer cells. Initial studies suggested that some isoforms of CD44 receptor, e.g., CD44v6 play important role in the process of metastasizing. Moreover, total amount of CD44 receptor detected with pan-CD44 antibodies was introduced as a marker of presence of tumour initiating cells regardless its splicing variability described later on [10]. Despite extensive research on CD44 and its isoforms (CD44v) its role as a marker of malignancy or patient’s prognosis remains unclear. However, it is clear that expression profile of CD44 changes as tumour develops. As mentioned above alternative splicing generates various isoforms of CD44 that appear and disappear at various stages of cancer development. This process of deregulated alternative splicing of CD44 starts already in adenomatous and hyperplastic epithelium.

CD44v isoforms play various roles in cancer that may have high or low impact on the key features of cancer cells like metastasizing or tumor initiating potential. It is concluded that tumors expressing certain isoforms of CD44v are more aggressive compared to tumors expressing CD44s-only [6]. CD44 isoform switching in cancer cells as well as normal cells happens in response to some stimuli like oxidative stress [11], activation of signal transduction pathways especially MAP kinases [12, 13], epigenetics [14] or upon activation of resting lymphocytes [15]. It is apparent that CD44 isoform switching mediated by alternative splicing of CD44 has an impact on epithelial-mesenchymal transition [16] and similarly on metastatic behavior of cancer cells [17–20].

CD44 has attracted unusual attention from scientist since it was shown that particular CD44v isoform appear in cancer cells during tumor progression. CD44v6 was one of the first isoforms that aspired to become a biomarker due to the studies showing its prometastatic effect in rats [20]. Moreover, anti-metastatic effect of antibodies raised against CD44v6 isoform supported CD44v6 as a putative biomarker of malignancy [18]. Later studies showed that the predictive role of CD44 and CD44v differs among tissues and that each tissue needs its own detailed study. Cancer stem cell model and a discovery of CD44 as a marker of tumor initiating breast cancer cells [10] further extended attractiveness of CD44. CD44 and its selected variants were further reported as markers of poor prognosis in gastric cancer patients. CD44v8-10 was later identified as a gastric cancer stem cell marker [21]. Generally, altered expression of CD44s and CD44v in various cancer types might have a certain prognostic value (see examples in Table 1).

**TABLE 1:** Clinical significance of various CD44 isoforms in certain cancer types. A reduced list of CD44 prognostic markers in various cancer types. met., metastasis; neg., negative.

Cancer type	Isoform	Clinical significance
Lung cancer	CD44v6, CD44s	Metastasis, survival [22, 23]
Breast cancer	Pan-CD44 (all isoforms)	Differentiation, neg. prognosis [10, 23]
Prostate cancer	CD44v6, ↓CD44s	Met., prognosis [23, 24]
Colorectal cancer	↓CD44s, CD44v6, CD44v9	Met., prognosis [23, 25, 26]
Bladder cancer	CD44v8-10	Neg. prognosis [27]

Fewer than 10% of colon adenomas progress to cancer [28]. Risk of progression to carcinoma is higher if villous architecture or high grade dysplasia is present. Colorectal carcinoma is preceded by pre-invasive stage of neoplasia that lasts for years and is typically asymptomatic [29]. Early detection of CRC could be possible through searching for biomarkers or markers of cancer cells [30].

In our study, we explored alternative splicing of CD44 directly in human precancerosis. We studied expression of variant isoforms of CD44 in precancerous tissue of the large intestine and compared the level of expression of CD44 in adjacent healthy mucosa in a cohort of 50 colorectal adenoma patients. We detected constant repetition of splicing and expression profile of CD44 receptor in the patients participating in our study. Our study puts CD44 receptor into the context of diagnosis of precancerosis rather than prognosis of CRC.

## Materials and Methods

### Cell Culture

Primary Human Epidermal Keratinocytes cells (Cell Applications, Inc.) were cultured in KGM-Gold™ medium with SingleQuots™ growth supplements (Lonza). HEL 12469—Human embryonic lung fibroblast (ECACC) were cultured in DMEM with 2 mM Glutamine (Healthcare Life Science HyClone) 1% Non Essential Amino Acids (GE Healthcare Life Science HyClone) and 10% Fetal Bovine Serum (GE Healthcare Life Science HyClone). The p53-deficient H1299 non-small cell lung cancer cell line was grown in RPMI-1640 medium supplemented with antibiotics and 10% FBS. Human breast carcinoma T47D cell line was grown in RPMI-1640 medium supplemented with antibiotics and 10% FBS. The cells were grown in a petri dish and detached at 80–90% confluency using accutase prior to incubation with HA-FITC.

### Endoscopic Screening and Sampling

Colonoscopy and mucosal sampling were performed in the Clinic of Internal Medicine—Hepatogastroenterology, University Hospital Brno using medical video endoscopes Olympus CF HQ190L, EVIS EXERA III. Endoscopic polypectomy and mucosectomy were proceed by standard technique using endoscopic needle, snare and electrosurgical unit (Interjet Needle Con 25G/4 mm/240 cm Boston, Dis Micro oval Snare 13 mm Boston, SD-990-15 HF polyp snare Olympus, VIO 300D ERBE). The tissue samples were collected using standard biopsy forceps (Radial Jaw 4 SC w/NDL, Boston). Samples for the purposes of our study were snap-frozen immediately after biopsy in isopropanol pre cooled on dry ice. This project is approved by the ethical committee of the University Hospital Brno. Each patient signed informed consent and participated voluntarily in our project. All experiments were performed in accordance with relevant guidelines and regulations.

### Real-Time and End-point PCR

Defrosted biopsy specimen was placed into 7 ml all glass Tissue grinder (Kimble Kontes). The homogenizer was then placed into liquid nitrogen for several seconds until the tissue froze and got fragile. The frozen tissue was then crushed using pestle B. The crushed tissue was then dissolved in lysis/binding buffer (Roche) and transferred into an eppendorf tube using PTFE tubing and 1 ml syringe. Total RNA from biopsy specimen was then isolated using High Pure RNA Tissue Kit (Roche, Cat. No. 12 033 674 001) according to the manufacturers protocol. Quality and concentration of isolated RNA was checked using NanoDrop2000 (ThermoFisher). Isolated RNA was then transcribed to cDNA using oligo dT primers (Invitrogen, Cat. No. 18418-020) and the M-MLV reverse transcriptase (Invitrogen Cat. No. 28025-013). End-point PCR was performed using Master Mix PCR, 2 mM (Generi Biotech, Czech Republic). 500 ng of cDNA and 0.25 μM concetration of the primers was used. Actin was used as a loading control. End-point PCR primers were as follows. CD44v3-c19: Forward 5′-AGG​CTG​GGA​GCC​AAA​TGA​AG-3′, Reverse 5′-TGG​GGT​GGA​ATG​TGT​CTT​GG-3′; CD44v4-c19: Forward 5′- ACG​GGC​TTT​TGA​CCA​CAC​AA-3′, Reverse 5′-TGG​GGT​GGA​ATG​TGT​CTT​GG-3′; CD44v5-c19: Forward 5′-CAC​ACC​CTC​CCC​TCA​TTC​AC-3′, Reverese 5′-GCT​CCA​CCT​TCT​TGA​CTC​CC-3′; CD44v6-c19: Forward 5′-GTG​GTT​TGG​CAA​CAG​ATG​GC-3′, Reverse 5′-GCT​CCA​CCT​TCT​TGA​CTC​CC-3′; CD44v7-c19: Forward 5′-GGA​CGA​GGT​CAT​CAA​GCA​GG-3′, reverse 5′-GGG​GTG​GAA​TGT​GTC​TTG​GT-3′; CD44v8-c19(8v8): Forward 5′-AAC​GCT​TCA​GCC​TAC​TGC​AA-3′, Reverse 5′-TGG​GGT​GGA​ATG​TGT​CTT​GG-3′; CD44v9-c19: Forward 5′-ACA​TCA​CAT​GAA​GGC​TTG​GA-3′, Reverse 5′-GCTCCACCT TCTTGACTCCC-3′; CD44v10-c19: Forward 5′-ACA​CGA​AGG​AAA​GCA​GGA​CC-3′, Reverse 5′- GCT​CCA​CCT​TCT​TGA​CTC​CC-3′; CD44s(CD44s1): Forward 5′-AGT​CAC​AGA​CCT​GCC​CAA​TG-3′, Reverse 5′-GCT​CCA​CCT​TCT​TGA​CTC​CC-3′; Actin: Forward 5′-AAT​CTG​GCA​CCA​CAC​CTT​CT-3′, Reverse 5′-AGC​ACA​GCC​TGG​ATA​GCA​AC-3′; Quantitative real-time PCR (qPCR) was performed using the LightCycler 480 instrument (Roche) and The LightCycler^®^ 480 SYBR Green I Master (Roche). 200 ng of cDNA and 0.1 μM concentration the primers was used for PCR reaction. Relative quantification of target genes expression was performed using the formula described elsewhere [31]. PCR primers were as follows. Actin: Forward 5′-AAT​CTG​GCA​CCA​CAC​CTT​CT-3′, Reverse 5′-AGC​ACA​GCC​TGG​ATA​GCA​AC-3′; 5 + v8 des3: Forward 5′-CCA​GTG​AAA​GGA​GCA​GCA​CT-3′, Reverse 5′-AGT​CCA​TAT​TGG​TAG​CAG​GGA-3′; 5 + 15 CD44s J4: Forward 5′-AAG​GAG​CAG​CAC​TTC​AGG​AG-3′, Reverse 5′-TGT​GTC​TTG​GTC​TCT​GGT​AG-3′; ESRP-1: Forward 5′-CAG​AGG​CAC​AAA​CAT​CAC​AT-3′, Reverse 5′-AGA​AAC​TGG​GCT​ACC​TCA​TTG​G-3′; ESRP-2: Forward 5′-GAC​GTG​CTT​GGC​TTC​CT-3′, Reverse 5′-AGC​TCC​TCA​CAA​GCA​AAG​AG-3′; Sam68: Forward 5′-ACC​GTA​AAC​CAG​TCG​GCA​TC-3′, Reverse 5′-CGT​CGT​CTC​CCC​ATT​CTC​AT-3′; TRA2B: Forward 5′-CTA​CGG​CGA​GCG​GGA​ATC-3′, Reverse 5′-GAC​TTT​GAT​CTG​GAA​CGC​CT-3′; SFRS6: Forward 5′-CTG​CGT​CGT​ATC​AAG​CGT​TTT-3′, Reverse 5′-TGT​ATC​AGC​CAG​TTG​ACT​CCG-3′;

### Western Blot Analysis

Whole cell lysis (WCL) buffer was used to dissolve crushed tissue. WCL formulation was 10 mM Tris at pH 7.4, 1 mM NaF, 1 mM Na_3_VO_4_, 1 mM PMSF, 0.3% SDS, 1% Triton X-100 and the protease inhibitor cocktail [32]. The crushed tissue was dissolved in WCL buffer and transferred into an eppendorf tube and sonicated on ice. The lysate was then spun down at 16,000 × *g* for 3 min, and the resulting supernatant was used for sample preparation by mixing with 2x or 4x concentrated Laemmli buffer and boiled for 4 min. An aliquot of the lysate was used for Protein level assay using BCA method (Pierce). Proteins were resolved by SDS/PAGE and transferred to PVDF membranes (GE Healthcare). After probing with a specific primary antibody and horse radish peroxidise (HRP)-conjugated secondary antibody, the protein bands were detected with the ECL kit using X-ray medical film (Kodak). The antibodies used were anti-actin IgG (AC-15, Sigma), anti-pan CD44 IgG (2C5 BBA10, R&D Systems), anti-CD44v10 IgG (AB2082, Millipore), anti-mouse IgG-HRP (A4416, Sigma), anti-rabbit IgG-HRP (NA934VS, GE Healthcare).

### Immunohistochemistry Staining—Fluorescent Detection

The snap-frozen tissues were sectioned to 8–10 µm slices using cryocut Leica 900 at −15°C and mount on gelatin coated histological slides. The tissue sections were then fixed using pre cooled acetone for 5 min and stored in the freezer for IHC analysis. The sections were defrosted in humidity chamber and washed 2 × 5 min in TBS 0.025% Triton-100. The sections were then blocked in 10% goat serum with 1% BSA in TBS for 1–2 h at room temperature. The slides were drained for a few seconds and wiped around the sections with tissue paper. Primary antibody was diluted in TBS with 1% BSA at 0.5 µl/100 µl and applied for 1 h. The slides were then washed 3x times in TBS 0.025% Triton-100. Fluorophore-conjugated secondary antibody diluted in TBS with 1% BSA at 1 µl/100 µl for 1 h was applied on the slide. 10 min before washing 100 µl of 0.3 µl DAPI/100ul TBS with 1% BSA was mixed in. The slides were then washed 3 × 5 min in TBS, mounting medium was added and coverslip placed. The slides were observed using epifluorescent microscope Nikon. We used anti-CD44 IgG (2C5, R&D Systems), goat anti-mouse FITC (SCBT).

### Flow Cytometry

The tissue samples used for HA binding test were stored in cold PBS prior to accutase extraction. Extraction of primary cells from adenomatous and healthy tissue was performed using the enzyme accutase. The tissue digest suspension containing colonocytes as well as stromal cells was left for 24 h in D-MEM serum free culture medium supplemented with Insulin 5 U/ml. The cells were then scraped off the petri dish using rubber policeman and washed twice with PBS. The cell suspension was then incubated for 15 min with fluorescently labeled Hyaluronic acid (HA-FITC, Sigma Aldrich) in serum free culture medium and finally washed once with PBS. The fluorescence intensity was then measured using flow cytometry (FACS Calibur, BD Bioscience).

### Statistics

Unless otherwise stated, data were analysed using the GraphPad PRISM 5.00 software, and represent mean ± SD of three independent experiments. Images are representative of at least three independent experiments. Relative quantification of target genes expression was performed using the formula described elsewhere [31]. Column statistics *t* test against hypothetical value of 1.0 was then used for overall statistical analysis of the whole group of patients.

## Results

Our previous research on CD44 receptor lead us to analyse alternative splicing profile (ASP) of CD44 gene in colon polyps. We originally observed a difference in ASP of CD44 between adenomatous and healthy colon mucosa in several tissue samples obtained from the University Hospital Brno. Consistent splicing differences between colon polyps and healthy adjacent mucosa throughout the pilot samples and the lack of exploration of ASP in colon precancerosis made us gradually extend the number of patients up to a cohort of 50 patients. The patients involved in our study were randomly selected during regular colonoscopy screenings. Fifty patients having conventional or serrated adenoma contributed with a biopsy specimen of their lesion and adjacent healthy mucosa (Figure 1B). Serrated adenoma *n* = 12, tubular adenoma *n* = 28, tubulovillous adenoma *n* = 10.

**FIGURE 2:**
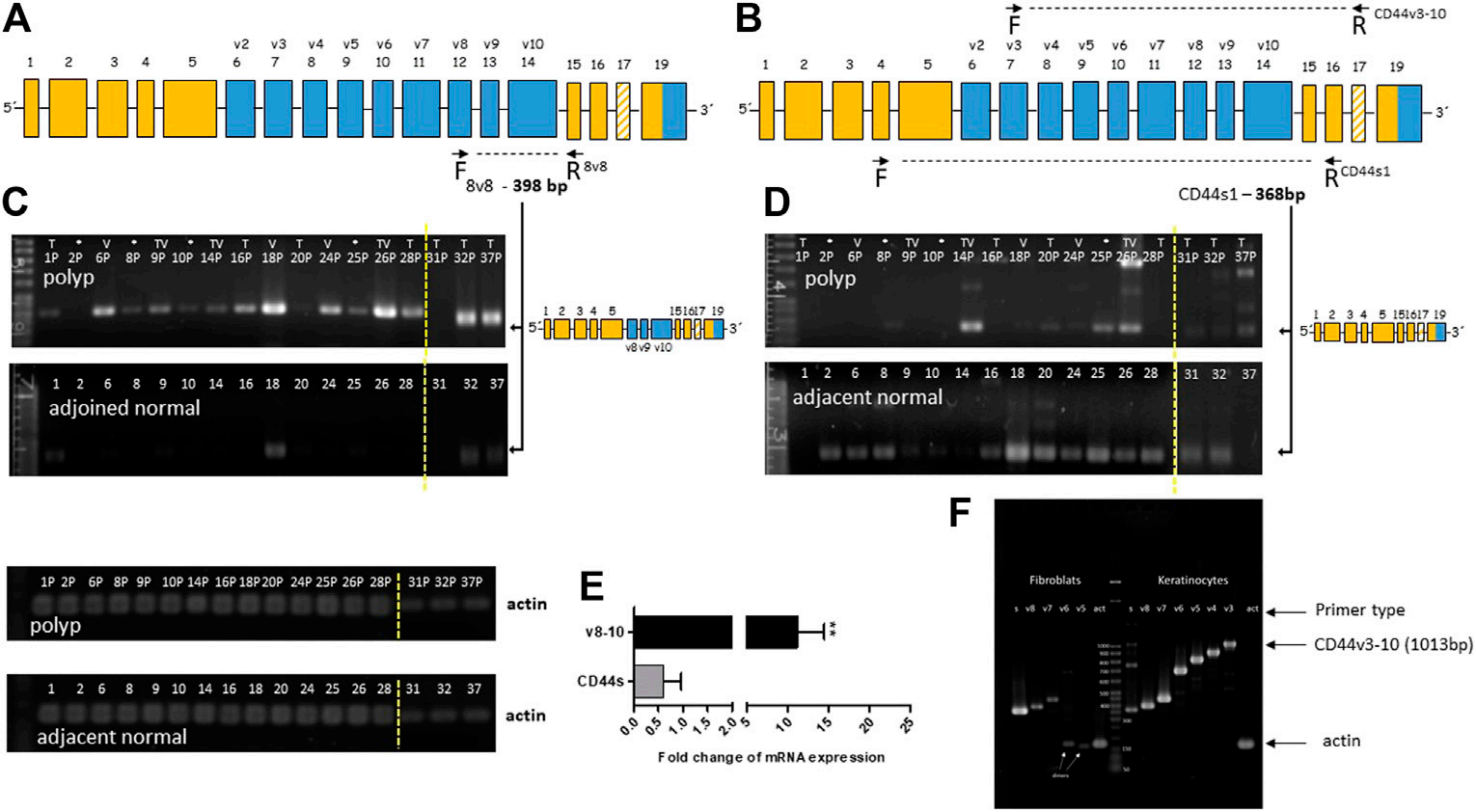
Overexpression of CD44v8-10 in colon polyps **(A,B)** Position of the selected primers in CD44 mRNA exon structure. Constant exons (always transcribed) are marked orange. Variable exons are marked blue. **(A)** 8v8 primers were used for detection of inclusion of variable exons v8-10, i.e., CD44v8-10. Product size of 398 bp confirms transcription of variable exons v8-10. **(B)** CD44s1 primers span the whole variable region of the CD44 gene. Product size of 368bp relates to the standard isoform of CD44 (CD44s). Product size >368 bp indicates inclusion of variable exons. **(C,D)** Agarose gel resolution of end-point PCR products of 8v8 primers **(C)** and CD44s1 primers **(D)** in polyp and healthy tissue samples. Actin was used as a loading control. PCR products for actin are displayed below. The arrows indicate product representing CD44v8-10 **(C)** and CD44s **(D)**. Relative quantification using densitometric analysis of agarose gels is represented by the bar graph **(E)**. An agarose gel after resolution of the PCR products of CD44v3 primers that start at exon v3 and finish at the constant region **(F)**. Keratinocytes were used as a positive control for exon inclusion while fibroblast express mostly CD44s. Increasing size of PCR products means higher number of variable exons included in the final mRNA transcript. Yellow dashed line delineates grouping of two gels into one picture. Samples from patients with sessile serrated adenoma (SSA) are marked with asterisk (*); samples of villous adenomas are marked (V); samples of tubular adenomas are marked (T); samples of tubulovillous adenomas are marked (TV).

We used end-point PCR to describe the overall alternative splicing profile (ASP) of CD44 in colon polyps and in adjacent healthy mucosa. PCR primers covered the whole variable part of the gene (marked blue Figure 2A,B). Verification of our end-point PCR set up was done using human primary keratinocytes that express all major isoforms of CD44v. We detected and displayed all major isoforms of CD44, CD44v (Figure 2F). We confirmed that our PCR method works well, identifies all major CD44v isoforms and is therefore a good indicator of aberrant splicing. Alternative splicing profile of CD44 in colon polyps differed substantially from alternative splicing profile of CD44 in adjacent healthy mucosa (Figures 2C,D). DNA electrophoresis showed that the main product of CD44s1 primers was size of 368 bp and corresponded to the standard isoform of CD44, i.e., CD44s was found in all samples of healthy tissue (Figure 2D). The same CD44s1 primers produced just a weak band of this size in less than 50% of polyp samples. Interestingly, products of higher size than 368 bp that respond to inclusion of variable exons were observed in polyp samples. We thus designed specific primers that coded products starting in particular variable region and ending in the constant region of CD44 gene, i.e., starting from exons v2-v10 and ending in C6, e.g., v8-C6 as shown in Figure 2A. We tested primary fibroblasts in order to have a negative control as well as to estimate any false positive contribution of fibroblast contamination to the level of variable isoforms in our lysates (Figure 2F). Agarose gels resolving PCR products of healthy tissue differed significantly from those resolving PCR products of polyp tissue. The gels representing polyp samples showed mainly PCR products of primers specific for v8, v9 and v10 exons while gels representing healthy tissue were blank. The molecular weight of PCR products confirmed theoretical product size of CD44v8-10 isoform. The end-point PCR data clearly indicated upregulation of CD44v8-10 isoform in polyp samples (Figures 2C,E).

**FIGURE 3:**
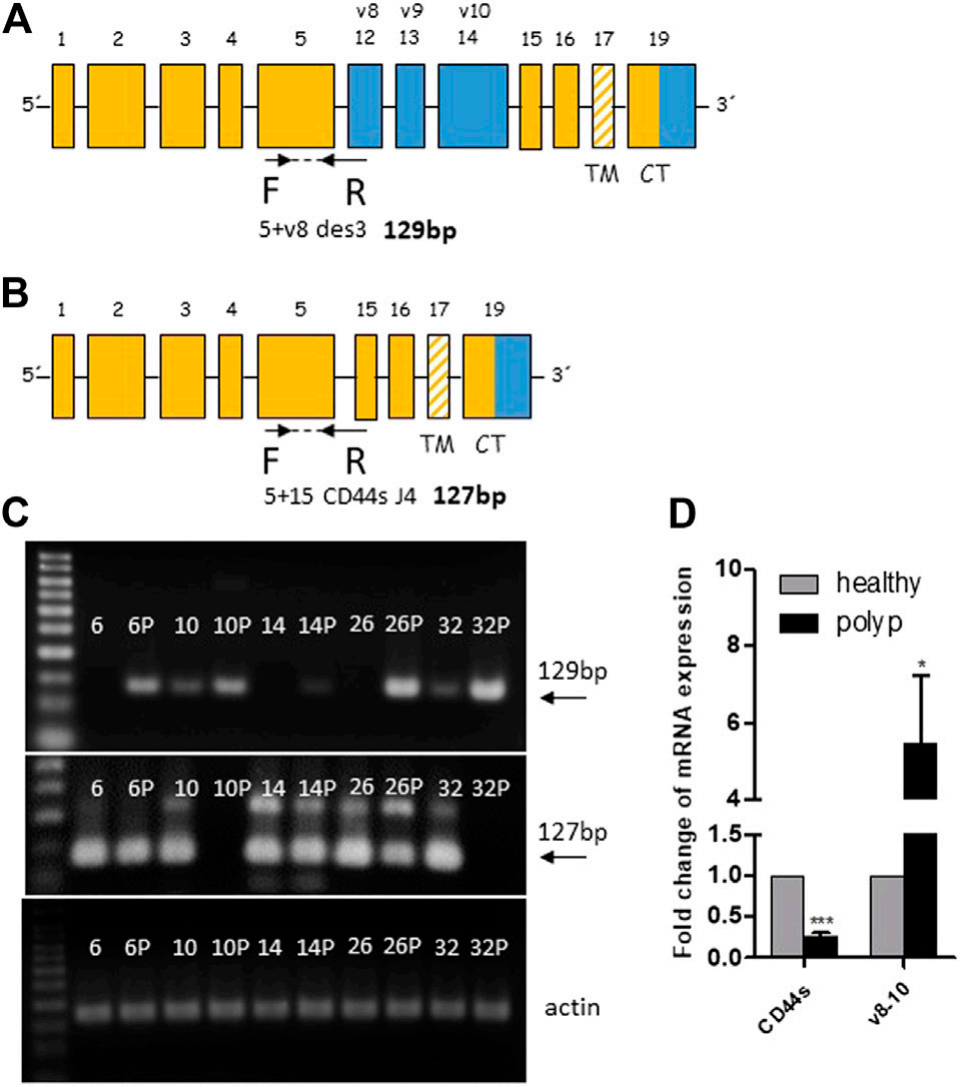
CD44v8-10 overexpression in colon polyps. Position of real time primers within the CD44 mRNA transcript of CD44v8-10 **(A)** and CD44s **(B)**. Reverse primers span exon junctions and anneal only if the following exon junctions occur C5+V8 **(A)** or C5+C15 **(B)**. Verification of appropriate size of PCR products was performed in random samples using 3% agarose gels **(C)**. Relative expression levels of CD44s and CD44v8-10 in colon polyps compared to adjacent healthy mucosa **(D)**. Statistical values are calculated using one sample *t* test. Column mean is significantly different from hypothetical value 1.0; *p* = 0.018 for v8-10; *p* < 0.001 for CD44s number of patients *N* = 50.

We subsequently performed real-time PCR to evaluate the overexpression of the polyp specific CD44v8-10 isoform. Previous end-point PCR experiments confirmed that v8 isoform is included only in v8-10 transcript. We designed primers spanning exon junction of constant exon C5 and variable exon v8 while antisense primer was complementary to the C5 constant exon (Figure 3A). The real time PCR analysis of 50 patients confirmed more than five-fold overexpression of CD44v8-10 isoform with *p* = 0.018 in colon polyps compared to adjacent healthy tissue (Figure 3D). 92% of patients had upregulated expression of CD44v8-10 in their polyps. Real-time PCR using primers specific for CD44s, i.e., primers spanning exon junction of C5+C15 Figure 3B revealed that CD44s is downregulated by twenty-fold in colon polyps compared to healthy tissue with *p* < 0.001 (Figure 3D). In other words, 100% of patients involved in our study had downregulated expression of CD44s in their precancerous lesions compared to their healthy adjacent counterparts. The size of real-time PCR products was also checked using agarose gel electrophoresis (Figure 3C).

**FIGURE 4:**
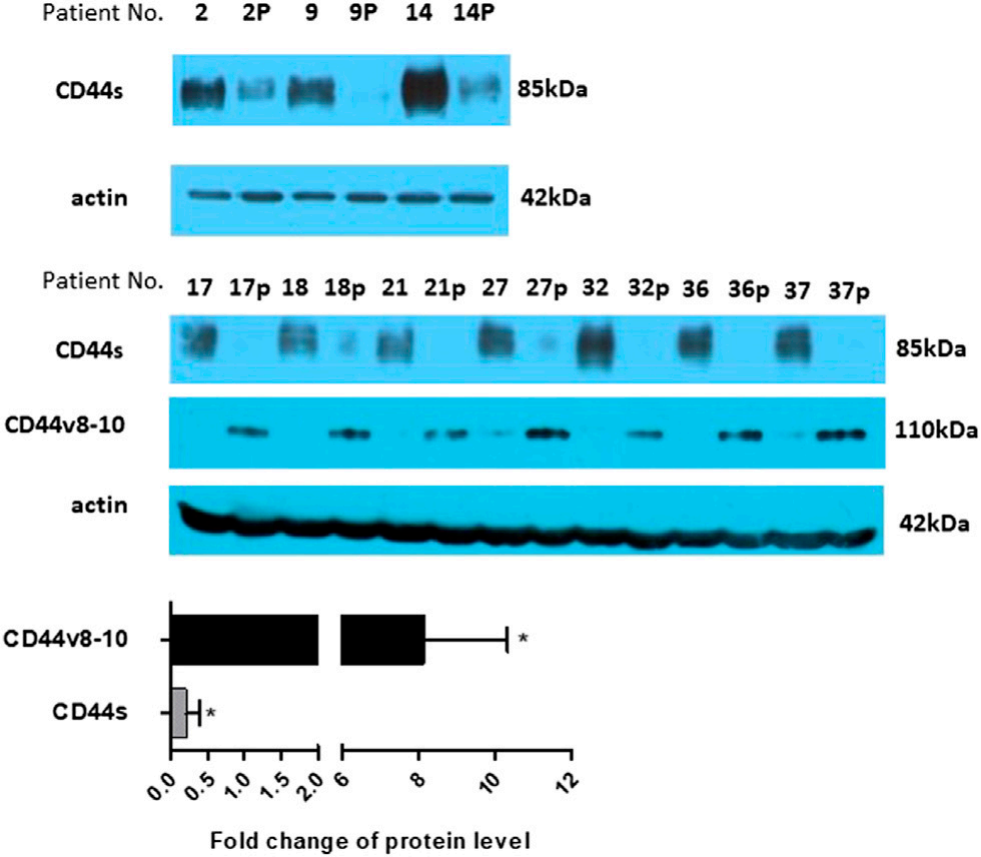
Protein expression. Expression of CD44s was checked on several larger samples using western blotting. The level of CD44s expression significantly drops in polyp samples. The level of protein expression was also estimated using densitometric analysis and put on a bar graph, bottom panel. Expression of CD44v8-10 is presented by bands at around 110 kDa. Actin was used as a loading control. “p” behind the number indicates polyp tissue.

Mouse anti-total CD44 IgG proved that CD44s is strongly downregulated also at the protein level in colon polyps (Figure 4). Not surprisingly, immunoblotting with rabbit anti-CD44v10 IgG that also recognizes CD44v8-10 [33] showed that polyp biopsies contain significantly larger amounts of CD44v8-10 isoform (Figure 4). This indicates that translation correlates with RNA expression.

**FIGURE 5:**
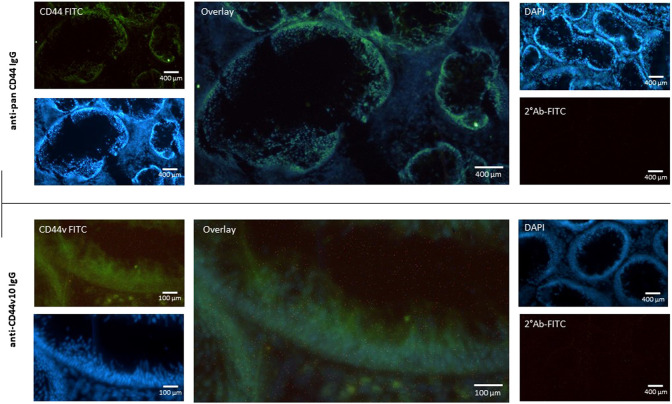
Expression of CD44 receptor in the mucosa of adenomatous polyp. Biopsy specimens of colon polyp were frozen sectioned and immunolabeled using pan specific anti-CD44 and anti-CD44v10 IgG. Nuclei were stained with DAPI (Blue). Nonspecific binding of secondary FITC IgG was checked (upper right panel). The data indicate that the most of CD44 protein is expressed in the epithelial cells of the colon crypt base.

To avoid any possible false positive results we decided for immunohistochemical staining of polyp tissue sections. It is known that fibroblasts express low levels of CD44v8-10 (Figure 2F). We identified the site of expression of CD44s and CD44v in tissue sections of colon polyps. The tissue sections were immunolabeled using combination of primary and fluorescently labeled secondary antibodies. The mouse anti-total CD44 IgG recognized all isoforms of CD44. Immunolabeling with mouse anti-total CD44 IgG showed that CD44 is mainly expressed in the epithelial lining of the colon (i.e., colonocytes) of both precancerous and healthy mucosa (Figure 5; upper panel). Counterstaining with DAPI and overlaying the images shows that fibroblasts and other cells contributing to colon mucosa do not express any significant amount of CD44 that could falsely affect our results that we ascribe to colonocytes. We also immunolabelled tissue sections with anti-CD44v10 IgG to see where CD44v8-10 localizes in colon polyps (Figure 5; bottom panel). The localization of CD44v8-10 was the same as demonstrated with anti-total CD44, i.e., in the epithelial lining of the colon polyps. Staining with secondary antibodies conjugated to FITC was used as a negative control of nonspecific interaction of the labeled antibodies with the tissues.

**FIGURE 6:**
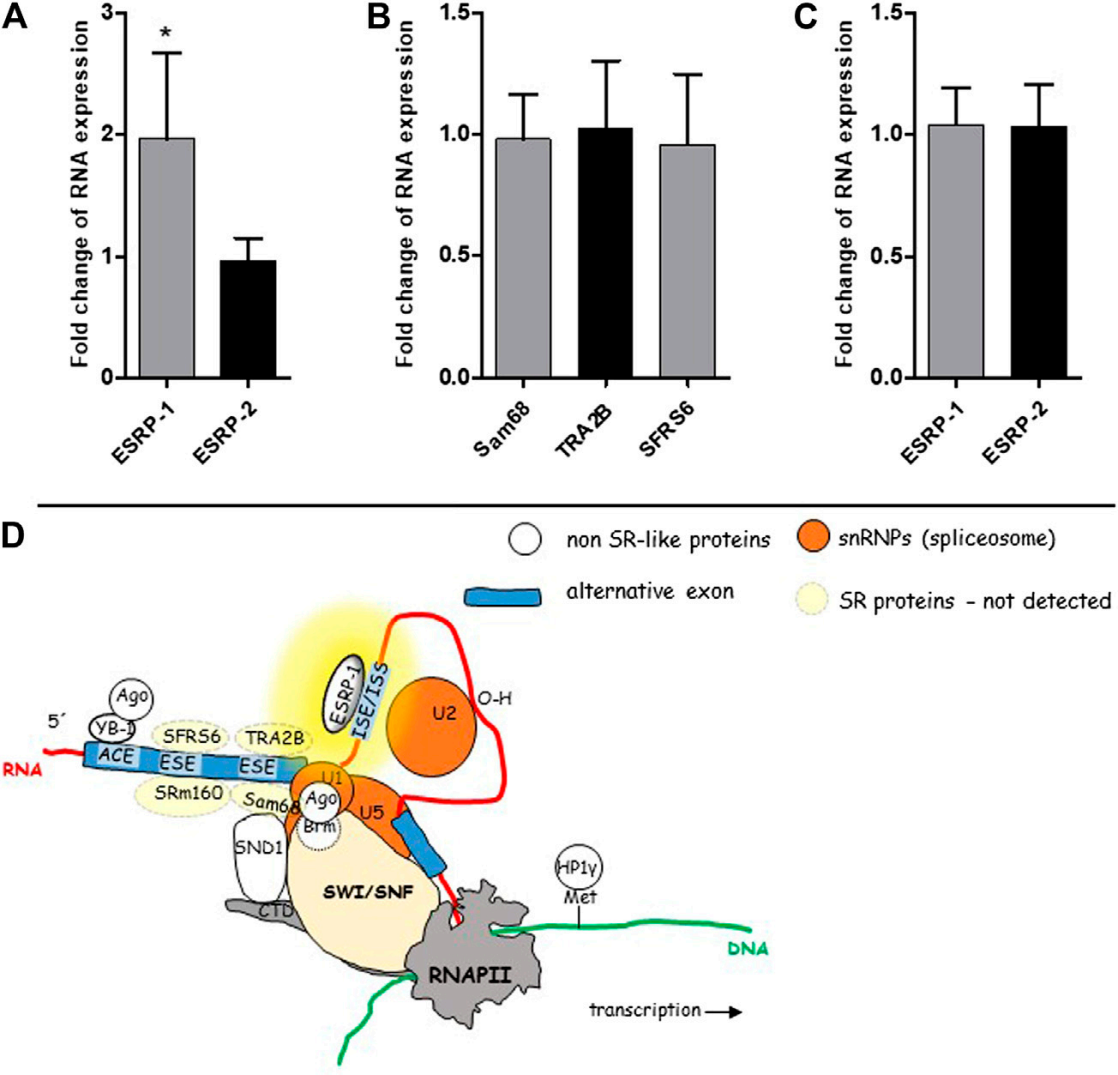
mRNA levels of some known splicing regulators. SR-like proteins and non SR-like ESRPs were tested in the cohort of 50 patients using real time PCR. ESRP-1 was almost two-fold overexpressed in colon polyps in 50 patients **(A)**. Expression level of ESRP-2 was similar in both types of colon mucosa. Relative expression levels of SR-like proteins in colon polyps compared to normal mucosa **(B)**. Relative expression levels of ESRP-1 and ESRP-2 in three patients whose polyps had the lowest rate of alternative splicing **(C)**. A graphical overview of suggested mechanism running in the background of ASP of CD44 in colon polyps **(D)**. The yellow background color highlights the site responsible for aberrant alternative splicing. The scheme was taken from our previous publication [6].

To shed more light on the mechanisms running in the background of our observation, i.e., overexpression of CD44v8-10 in colon polyps, we tested expression levels of RNA binding proteins ESRP-1 and ESRP-2 that are known to control ASP of CD44 in prostate epithelial cells [34] (Figure 6D). In conjunction with our previous work (Figure 6D), we also tested expression levels of SR-proteins SFRS6, TRA2B and Sam68 that are also known to affect alternative splicing of CD44 [6].

Only ESRP-1 gene was significantly upregulated in colon polyps (Figure 6A). We have not detected upregulation of ESRP-2 nor other SR-like proteins that have been previously documented to be involved in alternative splicing of CD44 gene (Figure 6B) [6]. Our data indicated that an ESRP-1 dependent isoform switching mechanism misprint might be running in the background of ASP in colon polyps. Not surprisingly ESRP-1 is not upregulated in colon polyps of those patients who express the lowest levels of CD44v8-10 (Figure 6C).

**FIGURE 7:**
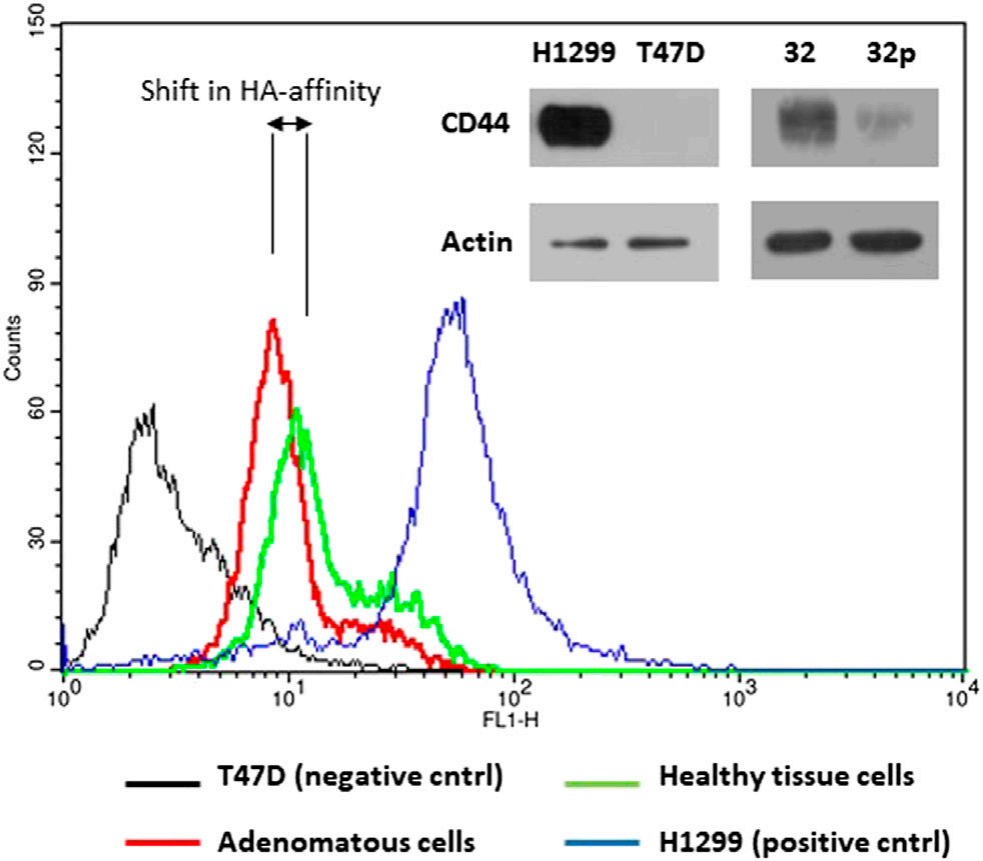
HA-FITC binds to CD44 receptor. The upper right panel of immunoblots shows CD44s protein expression in H1299, T47D cells and cells extracted from adenomatous (32p) and healthy colon (32) mucosa using anti-CD44 IgG and anti-actin IgG as a loading control. The part of the verification western blot was also published in Ref. 35. The histograms show fluorescence intensity of cells incubated with FITC labelled hyaluronan, natural ligand of CD44s receptor. CD44 non-expressing T47D cells were used as a negative control (black line); H1299 cells were used as a positive control for CD44-HA interaction (blue line); Cells extracted from the colon polyp (red line); cells extracted from healthy colon mucosa (green line). The histogram images are representatives of three independent experiments.

One of the main roles of CD44 receptor is its adhesion to extracellular matrix, mainly hyaluronic acid, HA. We tested affinity of the cells extracted from adenomatous and normal tissue samples to fluorescently labeled hyaluronic acid a natural ligand of CD44 receptor. Flow cytometry analysis showed higher fluorescence intensity of cells extracted from normal colon tissue compared to fluorescence intensity of cells extracted from colon polyps (Figure 7). We also used CD44 non expressing T47D cell line as a negative control and H1299 cell line as a positive control for HA binding as in our recently published study of targeting CD44 expressing cancer cells [35]. Our results show that aberrant alternative splicing of CD44 in colon polyps might affect affinity of colonocytes to HA, the main component of extracellular matrix.

## Discussion

The role of CD44 and its isoforms (CD44v) as markers of malignancy or patient’s prognosis remains unclear. Back in the early 90’s an early work of Wielenga et al. [36] reported higher expression of v7, v8-10, v6 and v5 isoforms throughout the progression of CRC [36]. Almost twenty ears later Kopp et al. [37] made a similar observation based on qualitative data, however immunohistology and protein level assays were missing. They remained the only ones who reported expression of CD44v8-10 in early adenoma. The authors did not relate their data to adjacent normal tissue neither suggested any mechanism running in the background of the process and thus the information is incomplete from our perspective. Moreover, the molecular processes in adenomatous mucosa were overshadowed by the main message regarding isoform switching during progression of CRC. Our study is strictly focused on precancerous tissues and compares splicing events in polyp and adjacent healthy mucosa within the same patient. We have revealed significant differences in alternative splicing of CD44 between healthy and precancerous tissue which is a novel observation. Alternative splicing of CD44 generates high numbers of variable isoforms. We used end point PCR in conjunction with agarose gel electrophoresis to positively identify expressed isoforms that became a subject of further research. We detected expression of CD44v8-10 isoform in all samples of colorectal adenoma biopsy specimen which is in accordance with a previous study [36] while only a few samples representing normal adjacent colon mucosa expressed CD44v8-10. The qRT PCR confirmed our observation, enabled quantification of the difference and allowed statistical analysis of the rate of overexpression of CD44v8-10 in colon polyps. Moreover, we proved that RNA splicing translates proportionally into the protein level, which might be a crucial condition for developing a protein based diagnostic approach. We used immunohistochemistry for identification of the site of origin of CD44 isoforms and RNA transcripts. Our data showed that all types of colorectal adenomas overexpress CD44v8-10 in the epithelial lining of the colon mucosa.

The majority of colorectal cancers arise via the conventional adenoma-carcinoma pathway while 10–20% of CRC arise from serrated lesions [38]. Serrated lesions follow a different pathway mostly due to microsatellite instability and BRAF/KRAS mutations. Conventional pathway is based on APC/KRAS/p53 mutation and chromosomal instability. Thus, serrated adenomas and conventional adenomas should be recognized as two biologically distinct subtypes of colorectal adenoma [39]. Diagnostic molecular markers should differ between these two subtypes. However, our data clearly indicate that v8-10 variant isoform of CD44 is an exemption and is present in each subtype of colonic precancerosis and therefore could be considered as a diagnostic marker of any type of colonic precancerous lesion if proper detection technique of CD44v8-10 is developed. We found overexpression of CD44v8-10 isoform in 92% of patients with polyps and strong downregulation of CD44s in all polyp samples which is in contrast to previous study [37] where CD44s was simply detected in every adenoma sample. This huge discrepancy between the two observations might be due to the fact that we quantitatively compared relative expression of CD44s in matched tissues while Kopp et al. qualitatively detected mRNA of CD44s without any quantification. Interestingly, only 36% positive fecal occult blood tests in the same cohort of patients were detected. The percentage of positive fecal occult blood tests within the cohort of patients participating in our study is similar to observations of others [40, 41]. This further highlights potential of CD44v8-10 as a diagnostic marker. CD44v8-10 has been already identified as gastric cancer stem cell marker [21]. Indeed, future of such diagnostic markers like CD44v8-10 seems to be very much predetermined and limited by available molecular diagnostic techniques. Detection of splice variants of CD44 in body fluids is already happening in several cancers and diseases like bladder cancer, endometriosis, gastric cancer and colon cancer [27, 42, 43] on the other hand detection of CD44 levels in healthy and oral SCC patients saliva and serum was found inconclusive [44]. Our future research will focus on possible detection of CD44v8-10 and CD44s in exfoliated cells isolated from stool.

Our data are in accord with a study of Zeilstra et al. who distinguished particular isoforms and where CD44v was shown to be important for intestinal tumorigenesis of mice while CD44s was not [45, 46]. It is unclear whether CD44s plays any role in intestinal tumor progression [47]. Our data indicate that finding and conclusions regarding the role of CD44 and its variable isoforms in intestinal tumorigenesis can be transferred into humans. Our data further support the notion that every isoform of CD44 plays different physiological role in the tissue specific manner and should be monitored. Importance of monitoring of isoform switching is further supported by studies where CD44 isoform status serves as a response prediction marker in CD44-HA interaction based therapy where CD44 antagonist (RG7356, Roche) is tested [48, 49].

We also show that polyp-origin colonocytes in the patient No. 32 have slightly lower affinity for HA, the major component of extracellular matrix probably due to altered expression of CD44s (Figure 7). It is also well known that CD44v8-10 isoform does not bind HA [50, 51]. We shed light on the processes running in the background of deregulation of alternative splicing of CD44 (Figure 6D). Non SR-like protein, ESRP-1, was suggested as a responsible regulator. This result is in accord with previous studies where ESRP proteins regulate variable exon inclusion into CD44 transcript [34]. We have set basis for the next set of studies focusing on mechanisms and applicability of our results in colon precancerosis screenings.

Our future research will focus on evaluation of diagnostic potential of CD44 and its isoforms mainly CD44v8-10 for early diagnosis of colon precancerosis and risk prognosis of colon cancer. Moreover, studies on isoforms of CD44 are of importance for development of hyaluronic acid targeted nanocarriers for anticancer drugs [35].

## Data Availability

The original contributions presented in the study are included in the article, further inquiries can be directed to the corresponding authors.

## Abbreviations:

ASP, alternative splicing profile; SR-protein, serine arginine rich RNA binding protein; ESRP, epithelial cell type specific splicing regulators; CD44s, standard isoform of CD44; CD44v, variable isoform of CD44.
